# Effectiveness and cost-effectiveness of stratified blended physiotherapy in patients with non-specific low back pain: study protocol of a cluster randomized controlled trial

**DOI:** 10.1186/s12891-020-3174-z

**Published:** 2020-04-22

**Authors:** Tjarco Koppenaal, Remco M. Arensman, Johanna M. van Dongen, Raymond W. J. G. Ostelo, Cindy Veenhof, Corelien J. J. Kloek, Martijn F. Pisters

**Affiliations:** 1 Department of Health Innovations and Technology, Fontys University of Applied Sciences, Eindhoven, The Netherlands; 2 Center for Physical Therapy Research and Innovation in Primary Care, Julius Health Care Centers, Utrecht, The Netherlands; 3 Physical Therapy Research, Department of Rehabilitation, Physiotherapy Science and Sport, Brain Center Rudolf Magnus, University Medical Center Utrecht, Utrecht University, Utrecht, The Netherlands; 4 Department of Health Sciences, Faculty of Science, VU University Amsterdam, Amsterdam Public Health research institute, Amsterdam, the Netherlands; 5 Department of Health Sciences, Faculty of Science, VU University Amsterdam, Amsterdam Movement Sciences research institute Amsterdam, Amsterdam, the Netherlands; 6 Department of Epidemiology and Biostatistics, Amsterdam University Medical Centre, Amsterdam, the Netherlands; 7 Research Group Innovation of Human Movement Care, HU University of Applied Sciences, Utrecht, The Netherlands

**Keywords:** E-health, Non-specific low back pain, Physiotherapy, Telemedicine

## Abstract

**Background:**

Patient education, advice on returning to normal activities and (home-based) exercise therapy are established treatment options for patients with non-specific low back pain (LBP). However, the effectiveness of physiotherapy interventions on physical functioning and prevention of recurrent events largely depends on patient self-management, adherence to prescribed (home-based) exercises and recommended physical activity behaviour. Therefore we have developed e-Exercise LBP, a blended intervention in which a smartphone application is integrated within face-to-face care. E-Exercise LBP aims to improve patient self-management skills and adherence to exercise and physical activity recommendations and consequently improve the effectiveness of physiotherapy on patients’ physical functioning. The aim of this study is to investigate the short- (3 months) and long-term (12 and 24 months) effectiveness on physical functioning and cost-effectiveness of e-Exercise LBP in comparison to usual primary care physiotherapy in patients with LBP.

**Methods:**

This paper presents the protocol of a prospective, multicentre cluster randomized controlled trial. In total 208 patients with LBP pain were treated with either e-Exercise LBP or usual care physiotherapy. E-Exercise LBP is stratified based on the risk for developing persistent LBP. Physiotherapists are able to monitor and evaluate treatment progress between face-to-face sessions using patient input from the smartphone application in order to optimize physiotherapy care. The smartphone application contains video-supported self-management information, video-supported exercises and a goal-oriented physical activity module. The primary outcome is physical functioning at 12-months follow-up. Secondary outcomes include pain intensity, physical activity, adherence to prescribed (home-based) exercises and recommended physical activity behaviour, self-efficacy, patient activation and health-related quality of life. All measurements will be performed at baseline, 3, 12 and 24 months after inclusion. An economic evaluation will be performed from the societal and the healthcare perspective and will assess cost-effectiveness of e-Exercise LBP compared to usual physiotherapy at 12 and 24 months.

**Discussion:**

A multi-phase development and implementation process using the Center for eHealth Research Roadmap for the participatory development of eHealth was used for development and evaluation. The findings will provide evidence on the effectiveness of blended care for patients with LBP and help to enhance future implementation of blended physiotherapy.

**Trial registration:**

ISRCTN, ISRCTN94074203. Registered 20 July 2018 – Retrospectively registered.

## Background

Low back pain (LBP) is the most common cause of disability in western society [1]. LBP causes a significant economic burden and is responsible for high direct healthcare costs as well as high indirect costs due to time lost from work [2]. LBP can be caused by a specific pathology or trauma; however, in more than 90% of cases an underlying disease is absent [3, 4]. The clinical course of this so-called ‘non-specific LBP’ varies; some people recover within a couple of days or weeks, and other people experience persistent disabling symptoms leading to chronic LBP [2, 5, 6]. Both national and international clinical LBP guidelines endorse patient education, advice on returning to normal activities and the prescription of home-exercises and/or supervised exercise therapy [7–10].

However, the effectiveness of physiotherapy in patients with LBP does not solely depend on providing the most adequate physiotherapy interventions. It also highly depends on patients’ adherence to prescribed (home-)exercises and recommended physical activity behaviour [11, 12]. Earlier research showed that 45–70% of patients do not adhere to prescribed exercises and physical activity recommendations [13–15], whereas adherent patients with LBP who continue a physically active lifestyle have a reduced risk of recurrent LBP [16]. Therefore, supporting self-management and adherence in patients with LBP is expected to be essential for the effectiveness of physiotherapy interventions on patients’ physical functioning and prevention of recurrent events.

Online applications, such as websites and apps, provide new solutions to support patients’ ability to manage their physical functioning in their home environment, and are promising to support self-management and adherence to prescribed (home-)exercises between face-to-face sessions [17–20]. Consequently, the integration of online applications into healthcare, so-called blended care [21], is expected to have several advantages for patients with LBP. Firstly, a blended intervention can stimulate self-management and exercise adherence to prescribed (home-)exercises and recommended physical activity behaviour in patients with LBP by its 24/7 online support and persuasive design [20, 22–24]. Secondly, the use of online applications enables monitoring and coaching of the patients’ individual health behaviour and provides the physiotherapist with information to optimize and tailor face-to-face care to the patients’ individual needs [22, 23, 25–27].

Despite all these benefits, matching the appropriate blended treatment for the individual patient is reported as a challenge [28]. To resolve this challenge within traditional face-to-face guidance, stratification tools have gained more attention in the last decade. Within a stratified-care approach, the treatment is matched upon the patients’ risk of developing persistent LBP, for example determined with the Keele STarT Back Screening Tool [29]. Research showed that such an approach results in improved physical functioning and satisfaction with care among patients with LBP while reducing costs of healthcare in both physiotherapy [30] – and primary care settings [31, 32]. Whereas the STarT Back Screening Tool can be used for matching the appropriate content of the face-to-face care to the individual patient, this tool also might have the same potential for matching the right digital content to the individual patient. Up until now, no other groups have used a stratification tool for personalization of blended physiotherapy as a whole.

Recently, the authors’ research group developed e-Exercise LBP, a blended and stratified intervention, in co-creation with patients, physiotherapists and experts [33]. E-Exercise LBP consists of face-to-face physiotherapy treatment, in which eCoaching is integrated using a smartphone application. E-Exercise LBP aims to improve patients’ physical functioning by offering a blended stratified-care approach, and consequently influencing patients’ self-management skills and adherence to exercise and physical activity recommendations in a positive way. At the long-term, e-Exercise LBP could result in an improved handling of recurrent LBP and direct and indirect costs. This blended care intervention is an adapted version of previously developed and evaluated blended physiotherapy programs [34, 35]. A pilot study using a prototype of the e-Exercise LBP intervention in 41 patients with LBP demonstrated feasibility and proof-of-concept on functional disability and pain [33]. Based on the results of the pilot study and end-user (patients and physiotherapist) usability experiences, the e-Exercise LBP program was further improved in preparation for the current study.

This study aims to investigate the short- (3 months) and long-term (12 and 24 months) effectiveness on physical functioning and cost-effectiveness of e-Exercise LBP, a primary care based personalized stratified blended care intervention, in comparison to usual primary care physiotherapy in patients with non-specific LBP.

## Method/design

### Study design

**Fig. 1:**
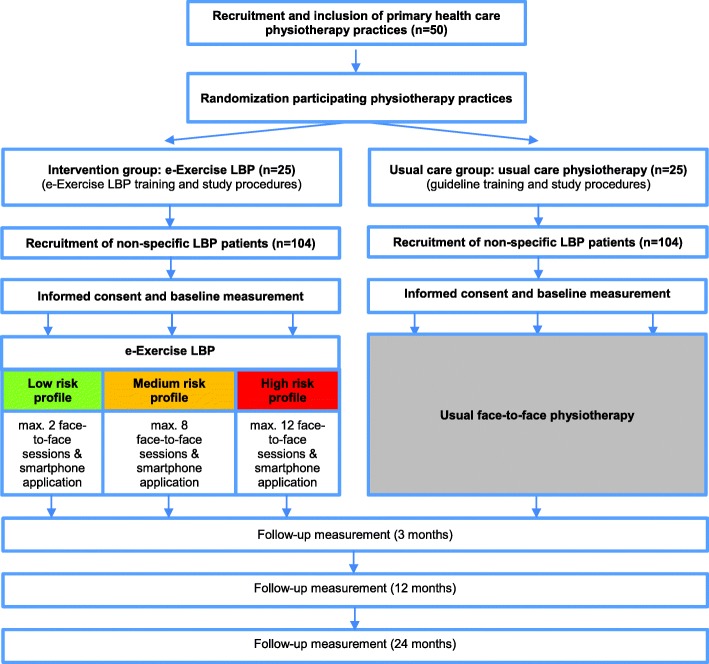
RCT study procedures

A prospective, multicentre cluster randomized controlled trial (RCT) will be conducted. The study has been approved by the Medical Research Ethics Committee of the University Medical Center Utrecht, the Netherlands (ISRCTN 94074203) for all centre sites. Within primary care, e-Exercise LBP will be compared to usual physiotherapy care. A flow diagram of the study protocol is shown in  Fig. 1.

### Participants

#### Primary care physiotherapy practices

Primary care physiotherapy practices will be invited by letter to participate in the study. Contact details of potential participating practices will be obtained from the professional network of the authors and a previous e-Exercise study [35]. Additionally, a recruitment advertisement will be placed in the online newsletter of The Royal Dutch Association for Physiotherapy (KNGF). Primary care physiotherapy practices are eligible to participate if at least five patients with non-specific LBP consult the practice for physiotherapy treatment each month. Each participating physiotherapy practice will be asked to enrol at least two physiotherapists in order to ensure continuity of care. All primary care physiotherapists, regardless of professional experience and education or specialization (e.g. manual therapist) are eligible to participate.

#### Patients

All patients with LBP who visit a participating physiotherapy practice will be invited to participate in the study.

Eligibility criteria of patients include: (i) being a patient requesting physiotherapy treatment for LBP, defined as pain in the lumbosacral region (sometimes associated with radiating pain to the buttock or leg) [10, 36], (ii) age 18 years or older, (iii) possessing a smartphone or tablet with access to the internet, (iv) mastery of the Dutch language.

Exclusion criteria include: (i) a specific cause of LBP determined through medical imaging or a medical doctor (e.g. osteoporotic fractures, spinal nerve compromise, malignancy, ankylosing spondylitis, canal stenosis, or severe spondylolisthesis), (ii) serious comorbidities (e.g., malignancy, stroke), (iii) current pregnancy, because of the prevalence of pelvic girdle pain as a specific form of LBP.

### Study procedure

Physiotherapy practices that are willing to participate in the study will be screened on eligibility by a researcher (TK or RA). Cluster randomization will be performed at the level of the participating physiotherapy practices. Each practice will be randomly assigned to the intervention (e-Exercise LBP) or the usual care group by an independent researcher using an a priori created computer-generated random sequence table. Physiotherapists in the intervention group will receive two 4-h training sessions about e-Exercise LBP and the study procedures. In the usual care group, physiotherapists will receive one 4-h training session in current best evidence practice according to the guideline LBP of the Royal Dutch Association for Physiotherapy (KNGF) [10] and the study procedures.

Physiotherapists, or their colleagues who will handle the initial registration of the patient, will orally inform potentially eligible patients about the study. Interested patients will receive the patient information letter by e-mail and will be contacted by one of the researchers (TK or RA) by phone prior to the first physiotherapy appointment. When a patient is willing to participate, a face-to-face appointment with one of the researchers (TK or RA) will be scheduled to screen in- and exclusion criteria and to provide written informed consent. After signing informed consent, the patient’s physiotherapist will be informed about the patient’s participation.

During the study period, both patient groups can still receive care from any other healthcare professional.

### Interventions

#### E-exercise LBP

A multi-phase development process based on the Center for eHealth Research (CeHRes) Roadmap [37] was used for development of the e-Exercise LBP intervention [33]. The e-Exercise LBP intervention integrates eCoaching using a smartphone application within face-to-face physiotherapy. The content is based on recommendations from national and international guidelines [7, 8, 10], and preferences and needs of patients and physiotherapists [33]. The principles of stratified care are used to personalize e-Exercise LBP to individual needs [30, 31].

##### Smartphone application

The smartphone application consists of three modules (Table 1): (i) An *information module* containing 12 weekly self-management themes (text and video), including assignments, about the aetiology of LBP, physical activity, patient experiences, pain management, and psychosocial factors related to LBP. (ii) An *exercise module* including a home-based video-instructed exercise program per prognostic risk profile. The selection, frequency and repetitions can be adjusted by the physiotherapist to address the patient’s specific functional limitations. (iii) A *physical activity module* containing a goal-oriented training program consisting of three sessions a week, to maintain or improve the level of physical activity for a self-chosen type of activity (e.g. cycling or walking). The training program starts with a 3-day baseline test, and can be optionally supported by graded activity functionality with tailored feedback, which was previously studied in two osteoarthritis studies [35, 38].

**Table 1:**
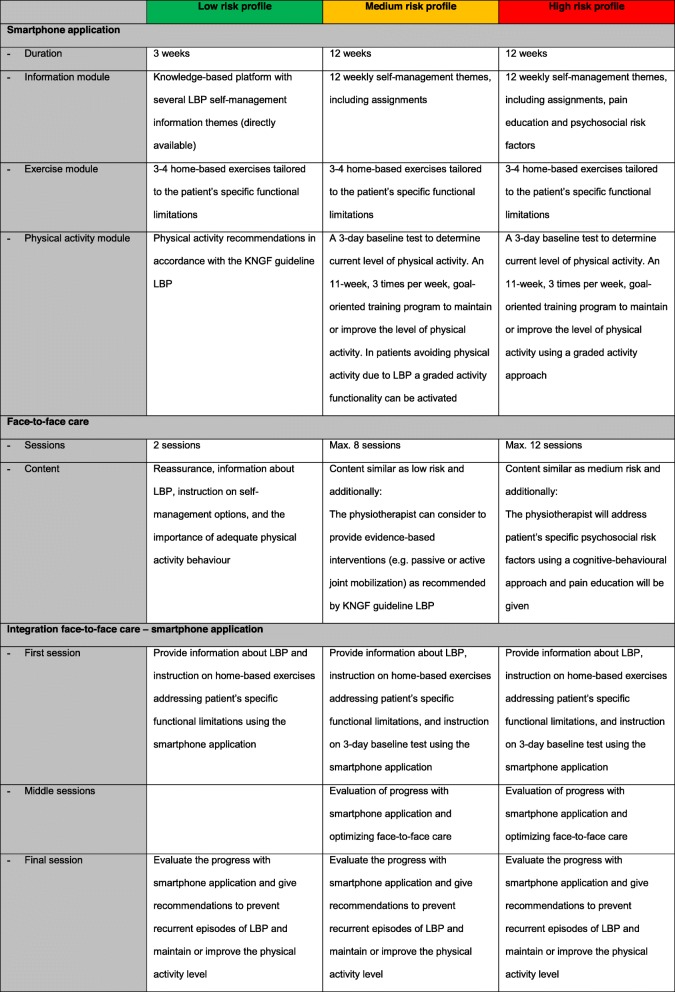
Overview e-Exercise LBP intervention

*LBP* Low back pain, *KNGF* Royal Dutch Association for Physiotherapy, *Max* Maximum

In patients having a “low risk” for developing persistent LBP the smartphone application will offer support for 3 weeks. In “medium” – and “high risk” patients the support will be 12 weeks. Afterwards the content of the smartphone application will remain available for the patients. In “low risk” patients the smartphone application will only contain the information – and exercise modules. In “medium – and high risk” patients the physical activity module will be added. The results of the baseline test of the physical activity module will be used by the physiotherapist and patient to set a goal to reach within 11 weeks. The graded activity functionality can be switched on in “medium risk” patients who avoid physical activity because of LBP. For “high risk” patients the graded activity functionality will always be activated. Print screens of the smartphone application are given in Additional file 1.

##### Face-to-face care

During the first face-to-face session, the physiotherapist will tailor the e-Exercise LBP intervention to the patients’ identified risk for developing persistent LBP (i.e. low, medium or high), using the Keele STarT Back Screening Tool [29, 39, 40] (Fig. 1, Table 1). Patients are asked to schedule their exercises and physical activities in the smartphone application, after which the smartphone application will sent automatic pop-up reminders accordingly. Physiotherapists will be able to monitor patients’ use of the smartphone application, monitor evaluated assignments, and select other types of exercises. With this information, the physiotherapist will be able to evaluate the progress and beliefs of the patients between face-to-face sessions, optimize the content of the smartphone application to patients’ individual needs, and tailor face-to-face care.

Physiotherapists are recommended to provide two face-to-face physiotherapy sessions to patients labelled as “*low risk*”, 8 sessions for patients labelled as “*medium risk*”, and 12 sessions for patients labelled as “*high risk*”. The objective of face-to-face care is to reassure the patient, provide information about LBP, instruct on self-management options, and underline the importance of adequate physical activity behaviour in accordance with the guideline LBP of the Royal Dutch Association for Physiotherapy (KNGF) [10]. Additionally, in medium- and high risk patients, the physiotherapist can consider to provide evidence-based interventions (e.g. passive or active joint mobilization) as recommended by the guideline LBP of the Royal Dutch Association for Physiotherapy (KNGF) [10]. In high risk patients, the physiotherapist will address patient specific psychosocial risk factors using a cognitive behavioural therapy approach, and pain education will be given [41, 42]. However, with respect to the physiotherapists’ clinical competences, physiotherapists are allowed to deviate from the e-Exercise protocol.

After completing e-Exercise LBP, the patient will receive fortnightly reminders from the smartphone application for up to 6 months to continue a physically active lifestyle.

#### Usual care

Patients in the usual care group will receive face-to-face usual care following the recommendations of the guideline LBP of the Royal Dutch Association for Physiotherapy (KNGF) [10]. Although eCoaching applications are not recommended in the guideline, physiotherapists from the usual care group are instructed to treat people without using any eCoaching applications. According to the guideline, the physiotherapy treatment includes information, exercises, and recommendations regarding physical activity. Practical content considerations will be made by the physiotherapists themselves with respect to their clinical expertise. The number of sessions will differ per patient.

### Measurements

Four time points (baseline, 3, 12 and 24 months) will be used for data collection of the primary and secondary outcomes using an online questionnaire. Baseline measurement will be conducted face-to-face and follow-up measurements preferably through online communication, e.g. Skype or FaceTime. When follow-up measurements through online communication are not possible, follow-up measurements will be conducted face-to-face. At all four time points participants will receive an accelerometer (Activ8) for the objective measurement of physical activity. Participants will be instructed to wear the Activ8 for five consecutive weeks at baseline and eight consecutive days at all following time points, except during sleeping, showering, bathing or swimming. For the economic evaluation, patients will be asked to complete eight retrospective 3-monthly online cost questionnaires. All of these questionnaires will have a 3-month recall period to cover the full duration of follow-up (i.e. 24 months). No financial incentives to complete questionnaires or to wear accelerometers will be offered. Table 2 gives a summary of all outcome measures and time points.

**Table 2:**
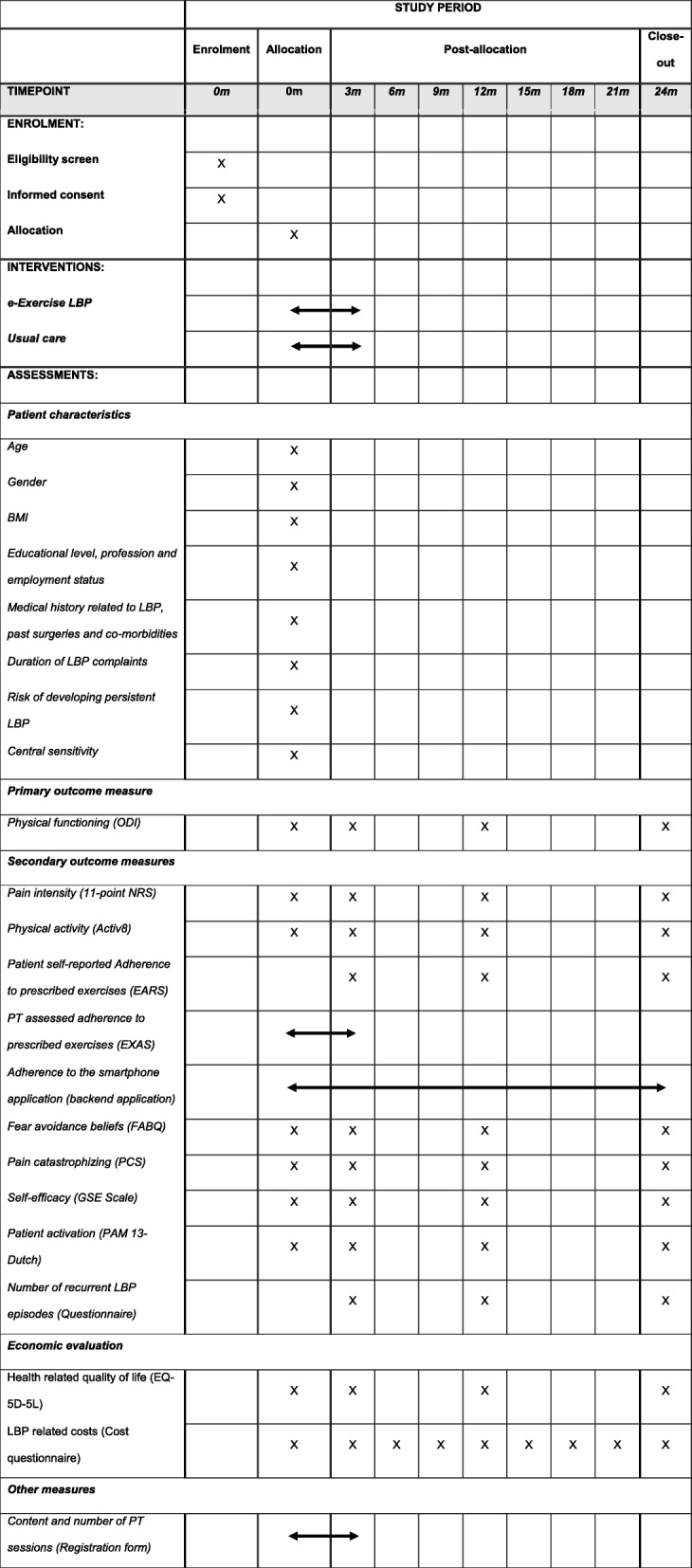
Schedule of enrolment, interventions, and assessments

Indicator for a period; duration of the period is not limited to length of the indicator and dependent on duration of interventions and use of smartphone application

*LBP* Low back pain, *BMI* Body Mass Index, *ODI* Oswestry Disability Index, *NRS* Numeric Rating Scale, *EARS* Exercise Adherence Rating Scale, *PT* Physiotherapist, *EXAS* Exercise Adherence Scale, *FABQ* Fear-Avoidance Beliefs Questionnaire, *PCS* Pain Catastrophizing Scale, *GSE scale* General Self-efficacy Scale, *PAM* Patient Activation Measure, *EQ-5D-5L* EuroQol 5D

### Primary outcome measure

The primary outcome measure is *physical functioning* and is derived from the internationally accepted “Core Outcome Set” (COS) for research into patients with non-specific LBP. The other recommended outcomes are included as secondary parameters, i.e. pain intensity, health-related quality of life, psychological functioning and pain interference [43–45] (Table 2). All selected measurement instruments in the current study are determined to be valid and reliable in previous research.

*Physical functioning* due to pain in LBP patients is assessed by the Oswestry Disability Index (ODI), version 2.1a [44–46].

### Secondary outcome measures

*Pain intensity* is measured with an 11-point Numeric Rating Scale (NRS) for the average LBP intensity in the last week [44, 45, 47].

*Physical activity* is objectively measured using a 3-axial accelerometer, the Activ8 (ACTIV8, Valkenswaard, The Netherlands) [48]. The Activ8 is a valid instrument to detect sedentary behaviour (combination of lying and sitting), standing, walking, running, and cycling. Additionally, MET-values are given. The Activ8 measures with 12.5 Hz, an epoch of 1 s a sample interval of 5 s. Every 5 min a summary is stored of the different postures and MET-values [49]. In addition, participants are requested to fill out a short activity diary about unusual activities and reasons for device removal.

*Patient self-reported adherence to prescribed home exercises* is measured by the Exercise Adherence Rating Scale (EARS). Besides that, the EARS measures the exercise prescription and the reasons for (non-) adherence [50].

*Physiotherapist based assessment of adherence to prescribed home exercises* is measured by the Exercise Adherence Scale (EXAS). The EXAS is an interview-based questionnaire which is used by the physiotherapist during face-to-face care to determine both the qualitative performance of the recommended home exercises and the agreement between recommended home exercises and patient reported adherence [51].

*Adherence to the smartphone application* is measured in the experimental group only by means of quantitative data about usage (e.g. completed modules). The data is automatically stored on the backend of the smartphone application.

*Fear avoidance beliefs about physical activity and work is* measured using the Fear-Avoidance Beliefs Questionnaire (FABQ). The FABQ assesses the fear of movement/(re)injury and consists of items related to physical activity and work [52].

*Pain catastrophizing* is measured by the Pain Catastrophizing Scale (PCS) The PCS is a self-report measurement tool that provided a valid index of catastrophizing in clinical and non-clinical populations [53, 54].

*Self-efficacy*, i.e. the patients beliefs in their efficacy to influence events that affect their lives [55], is measured using the General Self-efficacy Scale (GSE Scale) [56–58].

*Patient activation is* assessed by the Dutch version of the short form Patient Activation Measure (PAM 13-Dutch) [59, 60]. The Pam 13-Dutch assesses patient (or consumer) self-reported knowledge, skills and confidence for self-management of one’s health or chronic condition.

*The number of recurrent LBP episodes* is measured by the number of self-reported LBP episodes during the follow-up period. A recurrent LBP episode is defined as return of LBP with a minimum duration of 24 h after a period of at least 4 weeks without pain [61].

### Other measures

*Patient characteristics,* i.e. age, gender, educational level, profession, employment status, and medical history related to LBP over the past 2 years, are measured using an online questionnaire. Besides that, relevant clinical variables such as duration of current complaints, Body Mass Index, past surgeries, risk of developing persistent LBP, the presence of central sensitivity, and possible co-morbidities are collected.

*Content and number of physiotherapy sessions* are measured trough registration forms, developed by the researchers. The registration forms collect information on the number and content of face-to-face sessions, adherence to face-to-face sessions and deviations from the study protocol and are completed by the physiotherapists.

### Sample size calculation

The required sample size was calculated according to the recommendations of Campbell et al. (2010) for cluster randomized trials [62, 63], taking into account repeated measures of the primary outcome measure physical functioning on the ODI during follow-up [64]. An intracluster correlation coefficient of 0.05 was used [65, 66]. Additionally, to detect a clinical relevant difference between groups at 12 months following baseline, a difference of > 6 points in physical functioning on the ODI [67, 68] and a standard deviation of 14.5 [69] were used in the sample size calculation. For the repeated measures of physical functioning on the ODI a correlation of 0.5 is estimated between baseline and follow-up measurements until 12 months follow-up [64]. Based on these assumptions (using a power of 80% and α = 0.05) and average cluster size of 5, in total 165 patients are needed. With an expected dropout rate of 20% a total of 207 participating patients (104 patients per arm) are needed.

### Statistical analysis

Descriptive statistics (e.g. means and proportions) will be used to explore baseline comparability. To investigate selective attrition, general characteristics and primary baseline variables of dropouts and non-dropouts will be compared All analyses will be performed according to the ‘intention-to-treat’ principle. Missing data for all outcomes and cost measures will be imputed using ‘Multivariate Imputation by Chained Equations’ under the assumption that data are missing at random given baseline confounders. For all analysis, a two-tailed significance level of *p* < 0.05 is considered to be statistically significant. All analyses will be carried out using IBM SPSS Statistics version 24.0 (Amork, New York, USA).

#### Effectiveness

The primary purpose of this study is to estimate the effectiveness of e-Exercise LBP for improving physical functioning compared to usual primary care physiotherapy in patients with LBP. The primary analysis time point for the study will be 12 months following baseline, however 3- and 24-month changes will also be evaluated. To evaluate the overall effectiveness of e-Exercise LBP, differences in change scores per group and time period will be estimated on primary and secondary outcomes using linear mixed models (LMM) with random effects to control for correlation within patients and physiotherapists [70, 71]. The three-level hierarchy will exist of repeated measurements (level 1), nested within patients (level 2), nested within physiotherapists (level 3). Analysis will be controlled for baseline variables that have been shown to be related to physical functioning, e.g. age, gender, pain severity scores, duration of pain [72–74].

In addition, a per-protocol analysis that only includes patients of the intervention group which were adherent to the smartphone application and the entire usual care group will be performed. Patients will be considered to be adherent to the smartphone application if they used the application for at least 2/3rd of the duration (i.e. 2 out of 3 week for the “low-risk” profile and 8 out of 12 weeks for the “medium- and high-risk” profile) [35, 75]. Per-protocol analyses will be performed using LMM with the same 3-level structure, and will be controlled for the same variables as the primary analysis.

#### Economic evaluation

An economic evaluation will be performed from the societal and the healthcare perspective and will assess the cost-effectiveness of e-Exercise LBP compared to usual physiotherapy at 12 and 24 months.

##### Identification, measurement and valuation of costs

When the societal perspective is applied intervention, healthcare, informal care, unpaid productivity, and paid productivity costs will be included. When the healthcare perspective is applied, only costs accruing to the formal Dutch healthcare sector will be included. The costs of e-Exercise LBP will be estimated using a bottom-up micro-costing approach [76]. Information on the patients’ other kinds of resource use will be collected using eight 3-monthly retrospective cost questionnaires with 3-month recall periods. Healthcare utilization, unpaid productivity, and informal care will be valued in accordance with the “Dutch Manual of Costing” [77]. Paid productivity losses comprise of absenteeism (i.e. sickness absence) and presenteeism (i.e. reduced productivity while at work). Absenteeism was measured using a modified version of the IMTA Productivity Cost Questionnaire (iPCQ). Absenteeism will be valued in accordance with the “Friction Cost Approach” (FCA), using gender-specific price weights [78, 79]. Presenteeism will be measured using the “World Health Organization – Work Performance Questionnaire” as well as the “Productivity and Disease Questionnaire”, and valued using gender-specific price weights as well [78–81].

##### Measurement and valuation of health-related quality of life

The patients’ health states will be measured using the EuroQol-5D-5L (EQ-5D-5L) [82–85]. This questionnaire comprises of five health dimensions, i.e., mobility, self-care, usual activities, pain/discomfort and anxiety/depression. Per health dimension, patients are asked to indicate their severity level. Health states will be converted into utility values using the Dutch tariff [86] and Quality Adjusted Life Years (QALYs) will be estimated using linear interpolation between measurement points.

##### Statistical analyses

Missing cost and effect data will be imputed using ‘Multivariate Imputation by Chained Equations’ and the results will be pooled using Rubin’s rules [87]. Cost differences (∆C) and effect differences (∆E) will be estimated using LMM, and will be corrected for the same baseline variables as the effectiveness analysis. To account for the highly skewed nature of cost data, bias-corrected and accelerated bootstrapping with 5000 replications will be used to estimate 95% confidence intervals around the cost differences (∆C). Incremental cost-effectiveness ratios (ICERs) will be calculated by dividing the difference in costs by the difference in effects (∆C/∆E). Uncertainty surrounding the ICERs will be graphically illustrated by plotting bootstrapped cost-effect pairs on cost-effectiveness planes and by estimating cost-effectiveness acceptability curves. To test the robustness of the study results, several sensitivity analyses will be performed [88].

### Timeline

Recruitment of physiotherapy practices began in January 2018. The trial started in July 2018. Until January 2020 patients are able to enrol in the study. The follow-up will last until January 2022. Analysis of short-term effectiveness will start in March 2020, analysis of 12-month (cost-)effectiveness will start in January 2021.

## Discussion

This paper describes the design and methods of the e-Exercise LBP trial. The aim of the presented study is to investigate the short-term as well as the long-term effectiveness and cost-effectiveness of e-Exercise LBP compared to usual physiotherapy in patients with LBP. E-Exercise LBP is a stratified blended care intervention in which an eCoaching smartphone application is integrated into primary care face-to-face physiotherapy.

A major strength of this study is that the e-Exercise LBP trial is part of a multi-phase development and implementation process which was based on the Center for eHealth Research (CeHRes) Roadmap [37]. This holistic framework provides guidance during the participatory development of eHealth in order to enhance future implementation. As part of the development of the e-Exercise LBP intervention, needs and values of end-users and various stakeholders (e.g. physiotherapists, developers) were used to develop the first prototype [33]. Next, the prototype was tested on feasibility in a pilot study [33]. Based on experiences of patients and physiotherapists several important adaptations were made to the prototype of the e-Exercise LBP intervention. A first important adaptation is the development of a smartphone application, which was based on the web-based application used in the prototype. Secondly, the content of the smartphone application was stratified to match the stratification of face-to-face care for patients at low, medium or high risk for developing persistent LBP. As a result, the content of the smartphone application for low-risk patients was provided immediately instead of spread out over 12 weeks. The graded activity functionality was made mandatory for patients with a high risk for developing persistent LBP. On top of that, each information theme was enriched with an assignment in order to stimulate self-reflection. Overall, we believe that the improved smartphone application with various options for physiotherapists to personalize the content of the application, might help to improve patients’ level of physical functioning in patients with LBP.

Besides further development of the e-Exercise LBP intervention, several important methodological considerations were made with respect to the study design of the e-Exercise LBP trial. A first consideration was the use of a cluster-randomized controlled design to avoid contamination between the e-Exercise LBP intervention and usual physiotherapy care at the level of the participating physiotherapist. Cluster-randomization at the level of the participating physiotherapy practices ensures that each participating physiotherapist working in the same physiotherapy practice, delivers the same intervention [89]. The influence of clustering will be corrected using LMM in the statistical analysis.

Since the e-Exercise LBP intervention aims to improve physical functioning, this outcome measurement was selected as primary outcome measurement. Intervention duration will last up to 3 months, but a 12-month evaluation will provide insight in the effectiveness of e-Exercise LBP on the long-term. However, with respect to the cost-effectiveness, it is hypothesized that patients who followed e-Exercise LBP are able to manage recurrent complaints independently, resulting in reduced health-care usage or sickness absence. Since a 12-month follow-up might be too short to study this hypothesis, we added a 24-month follow-up focusing on the management of recurrent complaints.

Because the study design is well-considered, several potential operational issues are taken into account. An important operational issue is the physiotherapists’ training in the e-Exercise LBP intervention. From previous studies we learned that implementing a blended intervention into daily routine is a complex process that changes existing routines [28]. Therefore, training of the participating physiotherapists in the e-Exercise LBP intervention has been expanded from a 4-h training session to two 4-h training sessions. Additionally, Siilo, a secure messenger for healthcare professionals to communicate and share information, will be used during the study to be able to provide direct assistance to participating physiotherapists. And finally, instruction videos were created to support physiotherapists in using the e-Exercise LBP intervention. Another important operational issue is the possible increased risk of drop-outs during this study due to the 24-month follow-up period and the 11 questionnaires that have to be completed during this period. To minimize this risk, a researcher (TK or RA) will conduct the follow-up assessments at 3, 12 and 24 months in person, i.e. by phone, Skype or face-to-face. A final operational issue is the belief that e-Exercise LBP will not provide a solution for all patients having LBP, nor for all physiotherapists treating patients with LBP. Therefore, selection bias could occur, e.g. participants or physiotherapists having low digital literacy skills, or have a more negative attitude towards technology in general, are less likely to be included in this study.

However, with respect to our digitalized society it is expected that the majority of patients with LBP can benefit from the e-Exercise LBP intervention. The results of this study will help to understand whether blended physiotherapy for patients with LBP can be implemented on this basis.

## Supplementary information

**Additional file 1.** Print screens of the smartphone application.

## Data Availability

Not applicable as this is a protocol for a study.

## Abbreviations

CeHRes: Center for eHealth Research
COS: Core Outcome Set
EARS: Exercise Adherence Rating Scale
EQ-5D-5 L: EuroQol 5D
EXAS: Exercise Adherence Scale
FABQ: Fear-Avoidance Beliefs Questionnaire
FCA: Friction Cost Approach
GSE scale: General Self-efficacy Scale
ICERs: Incremental Cost-Effectiveness Ratios
iPCQ: IMTA Productivity Cost Questionnaire
KNGF: Royal Dutch Association for Physiotherapy
LBP: Low Back Pain
LMM: Linear Mixed Models
NRS: Numeric Rating Scale
ODI: Oswestry Disability Index
PAM: Patient Activation Measure
PCS: Pain Catastrophizing Scale
QALY’s: Quality Adjusted Life Years
RCT: Randomized Controlled Trial
