# Supplementary material for: Effectiveness and cost-effectiveness of stratified blended physiotherapy in patients with non-specific low back pain: study protocol of a cluster randomized controlled trial
Source: BMC Musculoskelet Disord. 2020 Apr 22;21:265. doi: 10.1186/s12891-020-3174-z (PMC7175532; doi:10.1186/s12891-020-3174-z)

**Additional file 1 – Print screens of the smartphone application**

**Information**


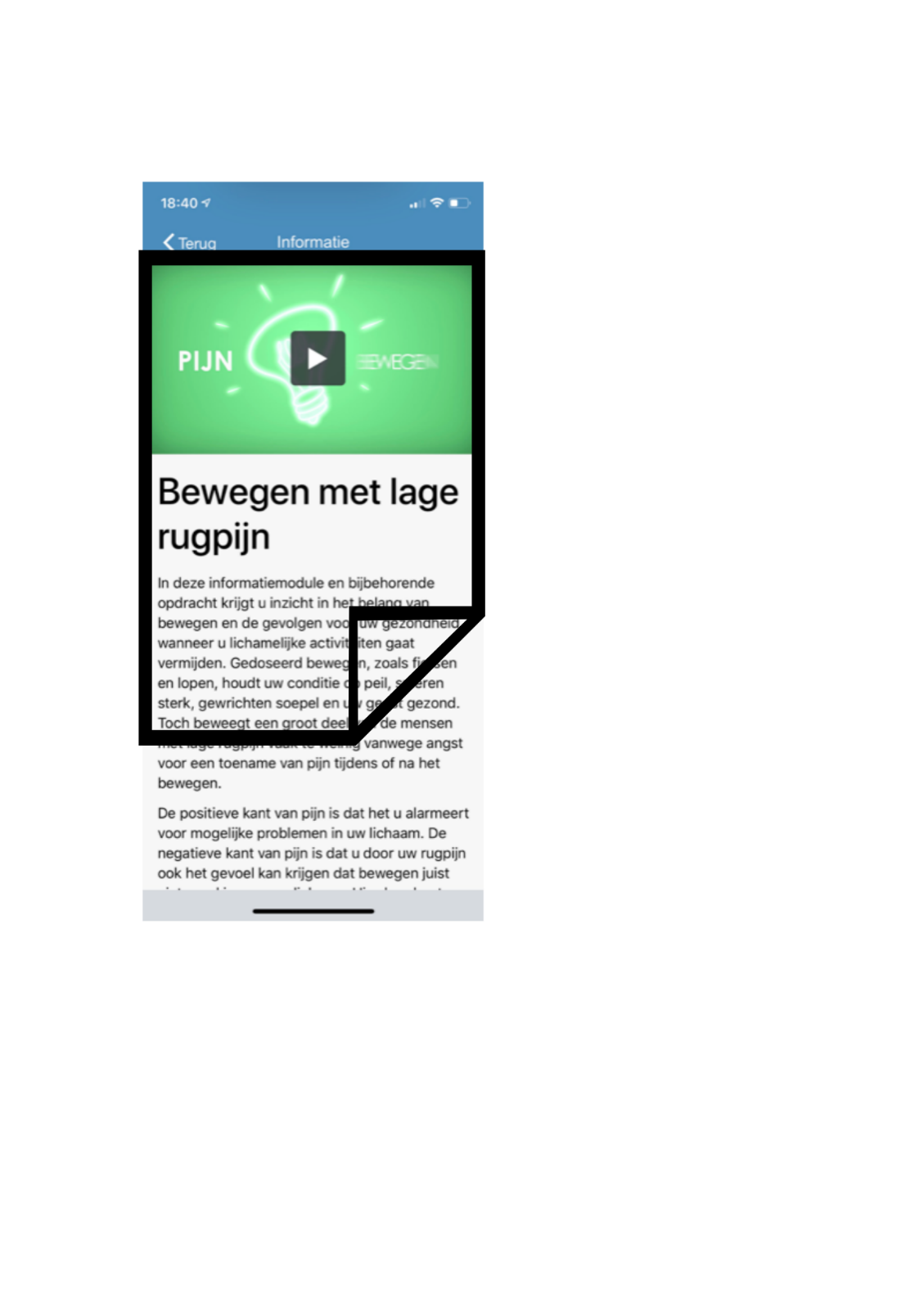

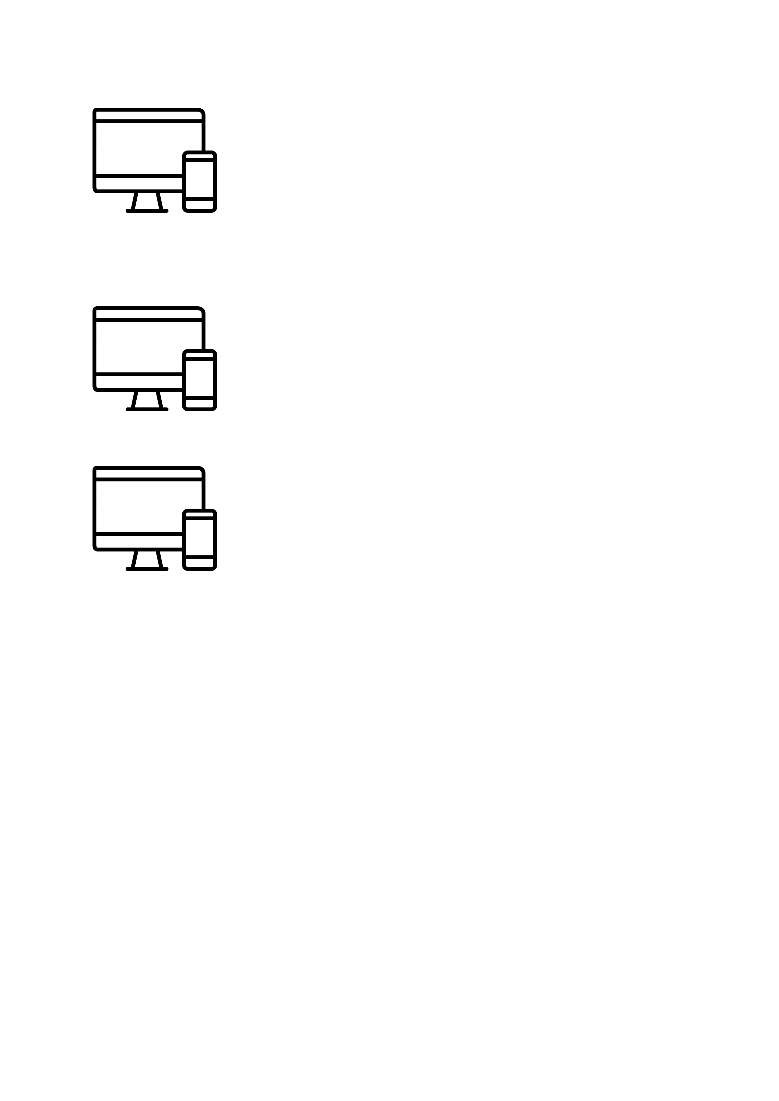


**Exercise**


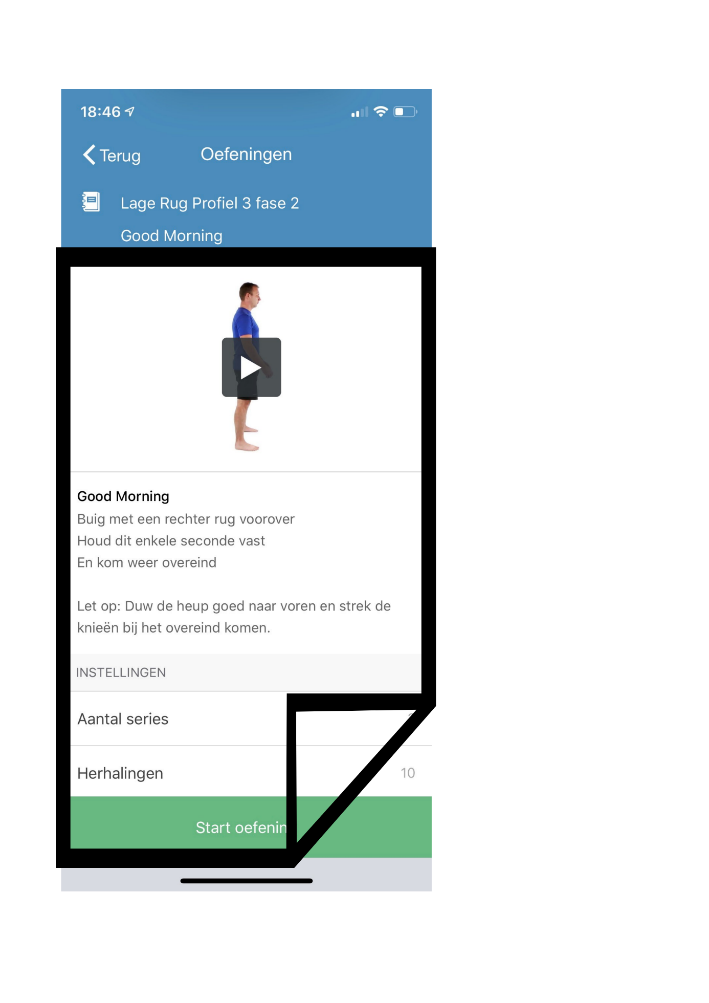

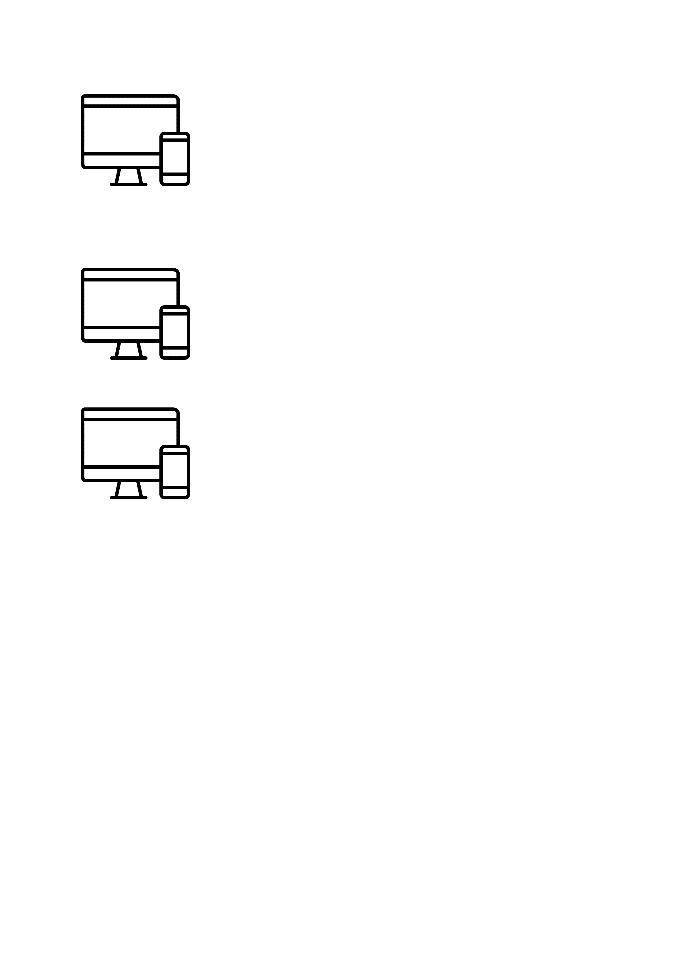


**Physical activity**


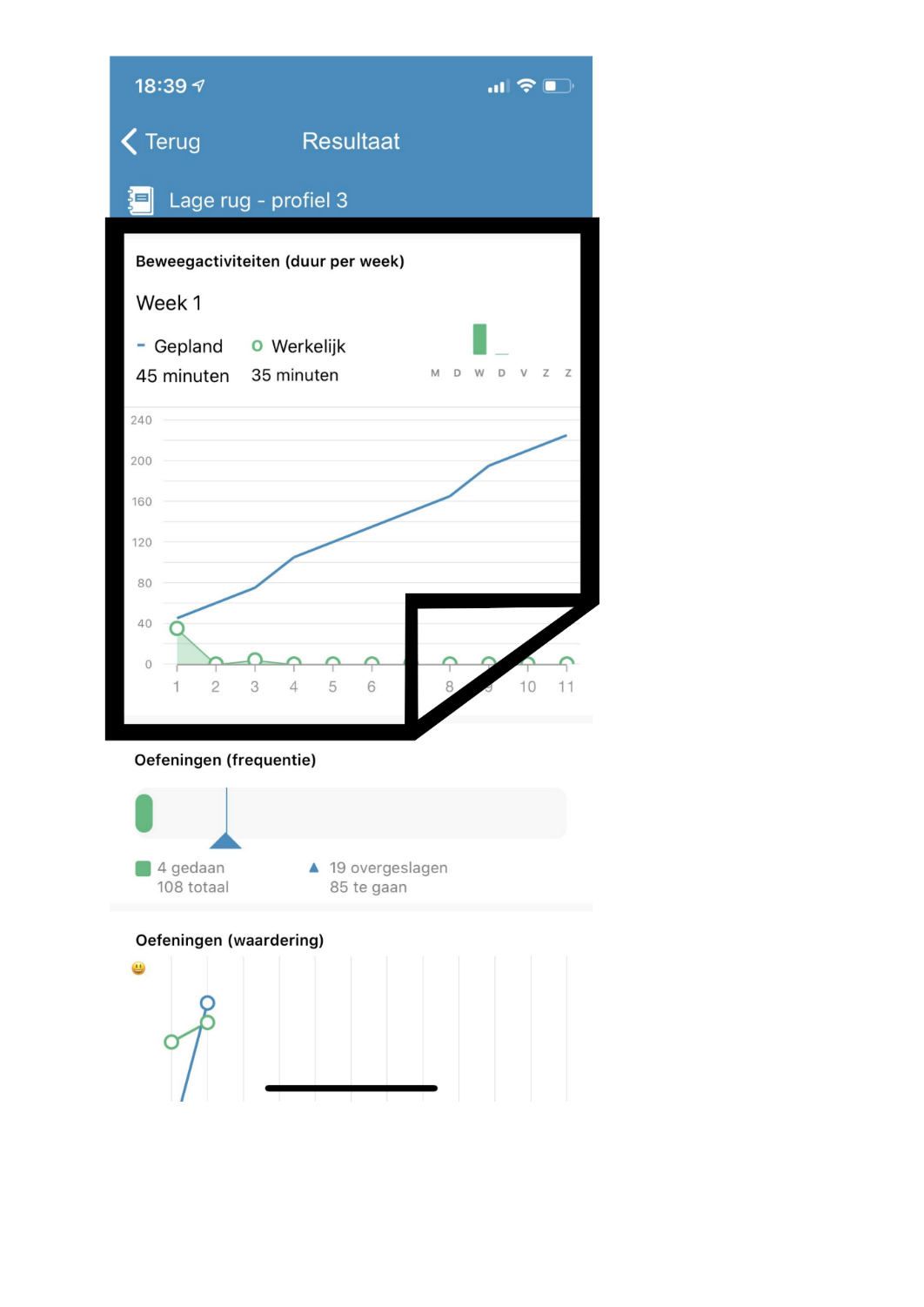

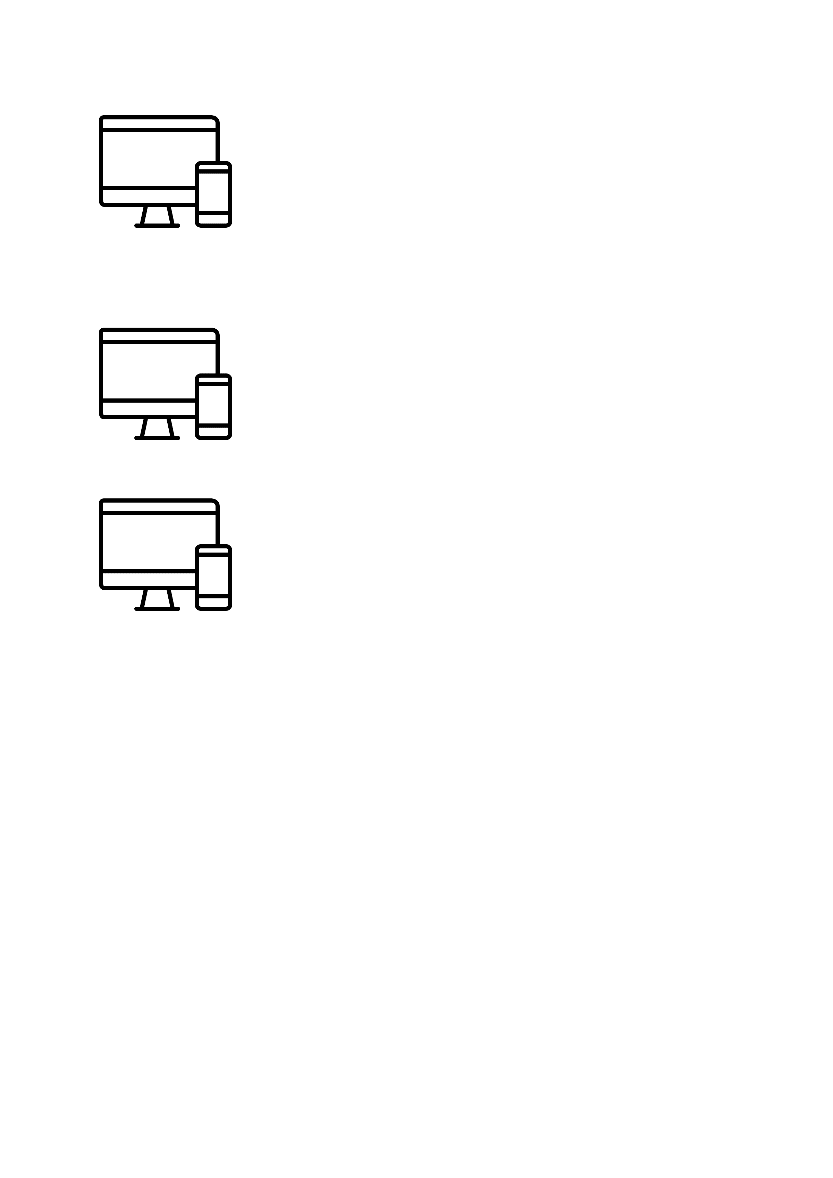

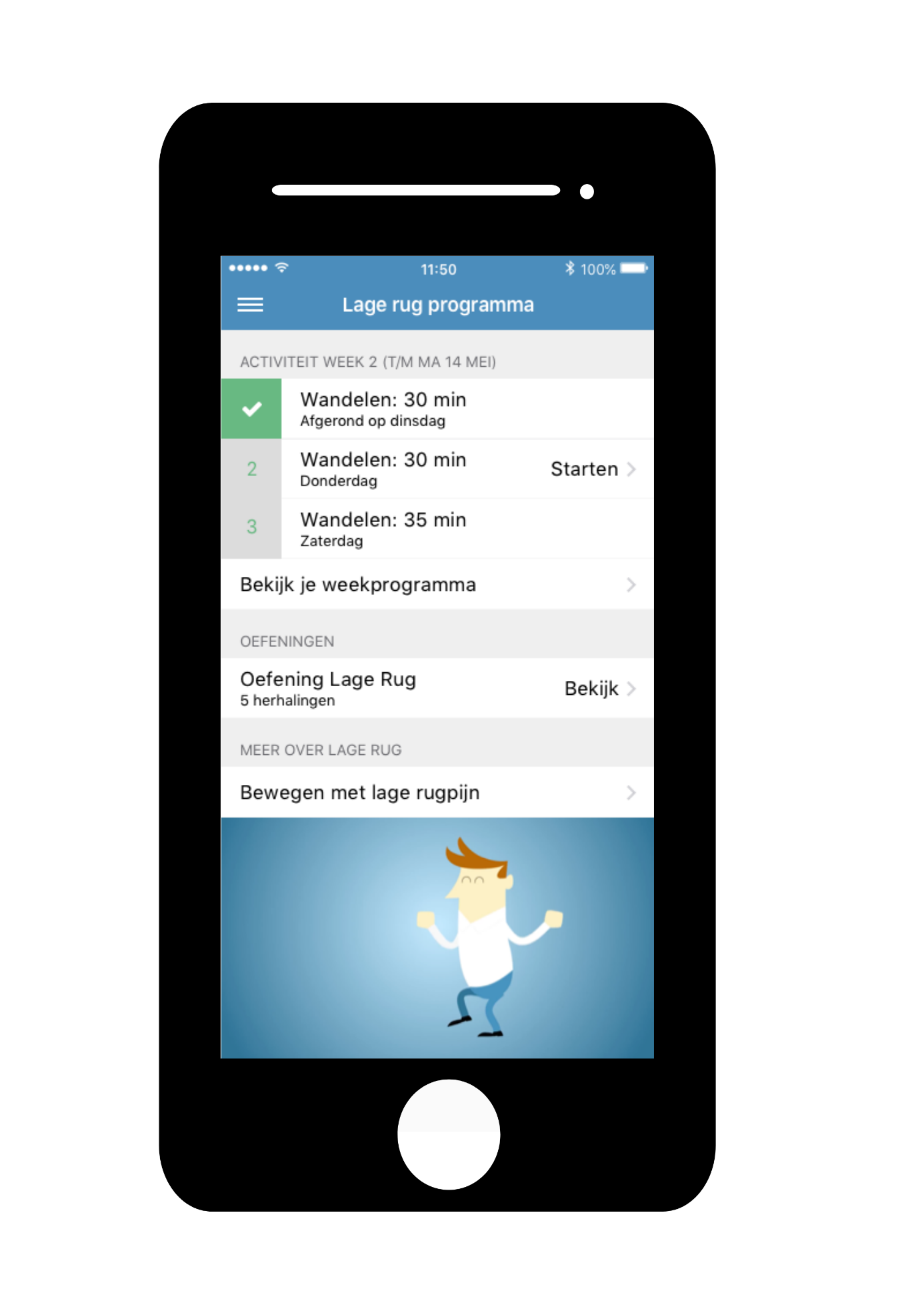

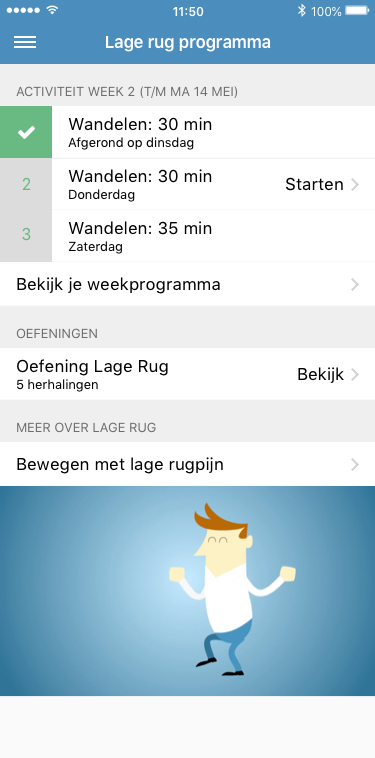

Supplement: Supplementary file 1 — Additional file 1. Print screens of the smartphone application. [file 12891_2020_3174_MOESM1_ESM.docx]
